# Programme science in action: lessons from an observational study of HIV prevention programming for key populations in Lusaka, Zambia

**DOI:** 10.1002/jia2.26237

**Published:** 2024-07-10

**Authors:** Izukanji Sikazwe, Maurice Musheke, Kanema Chiyenu, Benard Ngosa, Jake M. Pry, Chama Mulubwa, Martin Zimba, Martin Sakala, Mphatso Sakala, Paul Somwe, Goodwin Nyirenda, Theodora Savory, Carolyn Bolton‐Moore, Michael E. Herce

**Affiliations:** ^1^ Centre for Infectious Disease Research in Zambia (CIDRZ) Lusaka Zambia; ^2^ Department of Public Health Sciences School of Medicine University of California Davis California USA; ^3^ Zambia Sex Workers Alliance Lusaka Zambia; ^4^ Tithandizeni Umoyo Network Lusaka Zambia; ^5^ Intersex Society of Zambia Lusaka Zambia; ^6^ Division of Infectious Diseases Department of Medicine University of Alabama Birmingham Alabama USA; ^7^ Institute for Global Health and Infectious Diseases University of North Carolina Chapel Hill North Carolina USA

**Keywords:** programme science, key populations, HIV prevention, pre‐exposure prophylaxis, PrEP, Zambia

## Abstract

**Introduction:**

Optimizing uptake of pre‐exposure prophylaxis (PrEP) for individuals at risk of HIV acquisition has been challenging despite clear scientific evidence and normative guidelines, particularly for key populations (KPs) such as men who have sex with men (MSM), female sex workers (FSWs), transgender (TG) people and persons who inject drugs (PWID). Applying an iterative Programme Science cycle, building on the effective programme coverage framework, we describe the approach used by the Centre for Infectious Disease Research in Zambia (CIDRZ) to scale up PrEP delivery and address inequities in PrEP access for KP in Lusaka, Zambia.

**Methods:**

In 2019, CIDRZ partnered with 10 local KP civil society organizations (CSOs) and the Ministry of Health (MOH) to offer HIV services within KP‐designated community safe spaces. KP CSO partners led KP mobilization, managed safe spaces and delivered peer support; MOH organized clinicians and clinical commodities; and CIDRZ provided technical oversight. In December 2021, we introduced a community‐based intervention focused on PrEP delivery in venues where KP socialize. We collected routine programme data from September 2019 to June 2023 using programme‐specific tools and the national electronic health record. We estimated the before‐after effects of our intervention on PrEP uptake, continuation and equity for KP using descriptive statistics and interrupted time series regression, and used mixed‐effects regression to estimate marginal probabilities of PrEP continuity.

**Results:**

Most (25,658) of the 38,307 (67.0%) Key Population Investment Fund beneficiaries were reached with HIV prevention services at community‐based venues. In total, 23,527 (61.4%) received HIV testing services, with 15,508 (65.9%) testing HIV negative and found PrEP eligible, and 15,241 (98.3%) initiating PrEP. Across all programme quarters and KP types, PrEP uptake was >90%. After introducing venue‐based PrEP delivery, PrEP uptake (98.7% after vs. 96.5% before, *p* < 0.001) and the number of initiations (*p* = 0.014) increased significantly. The proportion of KP with ≥1 PrEP continuation visit within 6 months of initiation was unchanged post‐intervention (46.7%, 95% confidence interval [CI]: 45.7%, 47.6%) versus pre‐intervention (47.2%, 95% CI: 45.4%, 49.1%).

**Conclusions:**

Applying Programme Science principles, we demonstrate how decentralizing HIV prevention services to KP venues and safe spaces in partnership with KP CSOs enabled successful community‐based PrEP delivery beyond the reach of traditional facility‐based services.

## 1 INTRODUCTION

The mantra “leave no one behind” has taken on new meaning as countries in sub‐Sahara Africa (SSA) get closer to achieving global targets for ending AIDS [1]. Zambia, like other countries in SSA, has renewed focus on improving access to, and delivery of, HIV prevention services particularly among communities that have largely remained unreached, such as men who have sex with men (MSM), female sex workers (FSWs), transgender (TG) individuals and persons who inject drugs (PWID) [2]. HIV status awareness within these key populations (KPs) remains sub‐optimal, with rates as low as 22.6% for MSM and 60.5% for FSW [1], and HIV prevalence stubbornly high at 21% for MSM, 40% for FSW, 22% for TG and 15% for PWID [2, 3]. Addressing entrenched health inequities for HIV prevention and ensuring KP access to established HIV prevention technologies such as treatment as prevention and pre‐exposure prophylaxis (PrEP) is of utmost importance to reduce new HIV acquisition.

In 2016, Zambia adopted the 2015 World Health Organization PrEP recommendations with a once‐daily co‐formulated tablet of tenofovir disoproxil fumarate plus emtricitabine, as part of combination HIV prevention for individuals at risk of HIV acquisition [4, 5]. PrEP has slowly scaled up in Zambia, with 26,953 persons initiating PrEP in the first 2 years following its introduction [6], and an estimated 162,695 people receiving PrEP in 2022 [3]. PrEP is a cornerstone public health priority for the Zambian Ministry of Health (MOH) [7].

Despite recent advances in PrEP scale‐up, HIV prevention programming for KP in Zambia remains challenging due to cultural norms, religious beliefs, oppressive laws and other structural factors, including stigma and discrimination against KP. Self‐stigma, substance use and other individual factors further hinder access to HIV services for KP. These and other barriers hinder the real‐world reach and effectiveness of PrEP for KP. For example, while KP stand to benefit the most from PrEP, fewer than one‐third of all new PrEP initiators during the first 2 years of the Zambian PrEP rollout were KP [6]. By 2019, PrEP programming in Zambia remained hampered by ineffective demand creation strategies, limited KP‐friendly PrEP access points and support systems, and varying rates of beneficiary retention in PrEP programmes [6].

Programme Science is an approach that can be used to address service delivery gaps and inequities in PrEP provision and other areas of HIV prevention and public health programming. Oriented towards “getting research out of practice” and harnessing the wealth of information from routine programming to generate context‐specific knowledge on a continual basis [8], programme science is fundamentally concerned with issues of programme strategy, coverage and equity to maximize population‐level impact. While the concepts underlying programme science overlap with other disciplines, such as implementation science, it strives to move beyond conventional research processes [9].

In September 2019, the Centre for Infectious Disease Research in Zambia (CIDRZ), in partnership with KP civil society organizations (CSOs) and the Zambian MOH, launched a comprehensive KP programme through the PEPFAR‐funded Key Population Investment Fund (KPIF) [10] to overcome barriers to PrEP and increase access to quality HIV prevention, treatment, and care for MSM, FSW, TG and PWID. In this paper, we illustrate a pragmatic approach for applying Programme Science principles to improve KP PrEP programming [11, 12].

## 2 METHODS

### 2.1 CIDRZ programme science cycle

**Figure 1:**
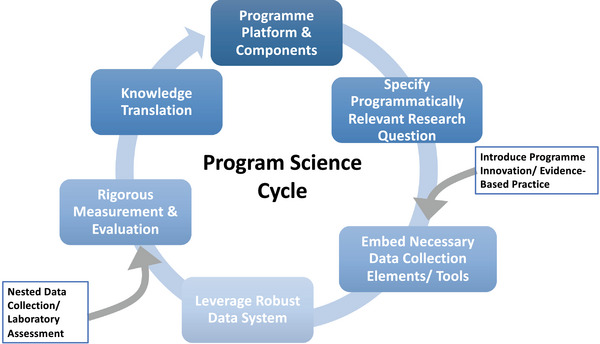
**CIDRZ Programme Science Cycle for generating health impact in Zambia**.

We applied insights from established programme science frameworks, including the effective programme coverage framework and Programme Science cycle [12], to crystalize our CIDRZ programme science approach and adapt it to the Zambian context [13]. We defined programme science similarly to others [11], with explicit emphasis on: improving service quality and equity; the central role of routine data systems and learning cycles to programme science practice; and community participation and data use in knowledge translation. We present a high‐level overview of the cyclic CIDRZ programme science approach in Figure 1.

During the CIDRZ programme science cycle, a research question and hypothesis is first stated at the programme planning phase [9]. We then leverage a robust national electronic health record (EHR), SmartCare, and programme indicators to answer the research question of interest, estimate the effects of our strategy or intervention on programme reach and impact, and produce context‐specific knowledge for Zambia that is then fed back to programme and community stakeholders for learning and programme refinement (Figure 1).

In this paper, we address the following *a priori* research question: What effect does decentralizing PrEP service delivery to community‐based venues and KP CSO‐managed “safe spaces” have on PrEP uptake, continuation and equity for KP in Lusaka, Zambia? Using this approach, and borrowing from established programme science frameworks [8], we describe our experience with PrEP decentralization and scale‐up for KP in four districts of Lusaka Province—Lusaka Urban, Chilanga, Kafue and Chongwe—where KPIF has been active.

### 2.2 Programme strategy overview: platform, population and components

KPIF is nested within the broader CIDRZ HIV prevention, care and treatment platform supported by PEPFAR through the US Centers for Disease Control and Prevention, and is a partnership among CIDRZ, KP CSOs and the MOH, operating at district, facility and community levels. The overarching goal is to increase access to, and uptake of, quality HIV prevention, care and treatment services for MSM, FSW, TG and PWID in Lusaka Province.

KPIF has several core programme components. First, it harnesses the intrinsic capacity of KP CSOs to lead service delivery and community engagement through peer navigation, community outreach and social network strategies (SNS). Second, KPIF partners with MOH trained government healthcare workers to provide KP‐friendly clinical services. Third, CIDRZ staff provide technical oversight and supportive supervision at all levels. Fourth, KPIF staff and partners draw upon their in‐depth knowledge of contextual factors, with, for example, KP CSO staff hailing from affected communities and MOH providers applying training on the unique needs, preferences and health‐seeking behaviours of KP to deliver health services. Fifth, KPIF utilizes differentiated service delivery models, such as KP CSO‐managed “safe‐spaces” situated within communities to reach KP with stigma‐free, person‐centred HIV services.

### 2.3 Programme component description

Before KPIF, PrEP services for KP were only nominally available in government health facilities. Travel distances, fear of stigma and discrimination, and for FSW, the opportunity costs incurred from long waiting times for health services, undermined PrEP uptake. To mitigate these challenges, at programme inception in September 2019, KPIF adopted a community approach, starting with empowering 10 KP CSOs to lead KP community mobilization activities to create demand for PrEP services. KP in need of PrEP were linked to health facilities and static KP safe spaces were established to provide HIV prevention and treatment services to KP. Rollout began with the introduction of PrEP in these KP‐designated static safe spaces in December 2019 in Chongwe and Kafue districts and then in April 2020 in the Chilanga district before expanding to the Lusaka Urban district in late 2021. The KP CSOs recruited, employed and trained peer navigators, who are KP community members themselves, to: engage in venue (“hot spot”) mapping and microplanning; offer peer health education; distribute condoms and lubricants; and collect basic programme data from KPIF beneficiaries.

KP CSOs used a combination of HIV prevention messaging and case‐finding strategies to reach KPIF beneficiaries. Specifically, these included peer navigation, SNS [14, 15]—a peer‐driven, chain‐referral strategy—and safe space testing to reach KP and supported distribution of HIV self‐test kits and index testing services [16]. KP newly identified as living with HIV were supported to initiate antiretroviral therapy offered by MOH at static KP safe spaces. KP testing HIV negative were referred to the safe spaces for a core combination HIV prevention package that included intimate partner violence screening and services, sexually transmitted infections screening and treatment, condoms and condom‐compatible lubricant, and PrEP. PrEP eligibility followed MOH guidelines [17], and was determined using the MOH‐approved screening tool, which asked about high‐risk behaviours in the last 6 months, including sexually transmitted infections, unprotected sexual intercourse with >1 partner and sharing injection equipment, among others. To encourage PrEP uptake and continuation, CIDRZ supported KP CSOs to form PrEP support groups managed by peer navigators.

### 2.4 Programme innovation: decentralized venue‐based PrEP delivery

In December 2021, in response to data revealing PrEP uptake being lower than KPIF targets, we pivoted to a more decentralized, venue‐based PrEP service delivery approach, with the goal of reaching more KP with PrEP through convenience of access. This meant offering PrEP *on site* at venues where KP socialize and/or meet new sex and/or needle‐sharing partners, such as bars and rest houses, rather than referring KP to centralized static KP safe spaces. All KP initiated on PrEP were assigned a unique identifier code to avoid double counting. KP CSO leaders met with community informants to identify potential KP venues. MOH provided supplies, such as HIV test kits and PrEP medications and KP‐friendly healthcare providers, including clinicians who delivered services at the venues. CIDRZ provided technical supervision, logistical planning and partner coordination to support venue‐level implementation. CIDRZ, MOH and KP CSO staff jointly conducted 288 KPIF venue‐based outreach events between December 2021 and March 2023.

### 2.5 Leveraging a robust data system

To assess programme outcomes, KPIF leverages CIDRZ monitoring and evaluation systems, KP CSO contextual knowledge and MOH data systems. KPIF monitoring and evaluation activities rely upon data recorded in routine MOH registers and the SmartCare EHR, programme‐bespoke data collection tools and KP CSO staff collecting data from beneficiaries. SmartCare is available at all KP safe spaces and supporting MOH health facilities. Together, these tools facilitate regular data quality management and real‐time monitoring of programme outputs and outcomes.

### 2.6 Rigorous measurement and evaluation

In this analysis, the evaluation of the PrEP programme impact is aligned with PEPFAR indicators for Monitoring, Evaluation, and Reporting (Indicator Reference sheet, versions 2.5 and 2.6) [18]. These indicators include variables for HIV testing and HIV test results, and describe each step of the PrEP cascade. These variables capture: the number of KPs reached with individual and/or small group HIV prevention interventions, including HIV testing; and the number and proportion of KPs who test HIV negative who have been screened for PrEP, found eligible for PrEP, newly enrol on PrEP and return for a follow‐up or re‐initiation visit to receive PrEP.

#### 2.6.1 Study outcomes

For this analysis, we examine *a priori* outcomes of programme reach, PrEP initiation and PrEP continuation. We defined PrEP initiation and continuation as documented evidence of a first time or subsequent (within 6 months of initiation) PrEP medication collection, respectively. We devised a *post‐hoc* outcome to assess the share of all PrEP initiators who were KP, defined as the number of PrEP initiations among KP divided by the total number of PrEP initiations within the 18 facility catchment areas where PrEP programming was offered by *both* MOH in facilities and KPIF in the community. We compared the proportional share of PrEP initiations among KP versus general population members before and after the introduction of venue‐based PrEP delivery to examine equity in PrEP access for KP.

#### 2.6.2 Statistical analysis

We analysed de‐identified line‐listed PrEP data from KPIF inception in September 2019 (i.e. quarter 3, Q3 2019) through June 2023 (Q2 2023). We defined the date of introduction of our programme innovation as 1 December 2021. We tabulated numerical counts and calculated proportions, frequency distributions and summary statistics. We used Pearson's Chi‐squared test to examine associations with programme outcomes before and after the introduction of our venue‐based PrEP innovation, and with KP group, district and age band. We conducted a sensitivity analysis using interrupted time series regression to account for variations in outcome due to calendar time [19]. We used mixed effects regression to develop marginal probability plots. All analyses were performed using Stata (StataCorp, Austin, TX, v16.3).

### 2.7 Ethics statement

The study was approved by the U.S. Centers for Disease Control & Prevention (#2019‐201), University of Zambia Biomedical Research Ethics Committee (#005‐11‐17) and the University of North Carolina IRB, USA (#18‐1060) without requiring consent for review of de‐identified, routinely collected data.

## 3 RESULTS

### 3.1 Overview

We first describe KPIF population demographics and beneficiary flow from programme enrolment through PrEP initiation and follow‐up. We then present data for KP initiating PrEP by programme quarter and KP group and examine trends in PrEP continuation. Finally, we compare PrEP uptake between KP and the general population in catchment areas where KPIF was active.

### 3.2 KPIF population

The 38,307 unique KPIF beneficiaries reached with HIV prevention messaging were comprised mostly of people born female at birth (54.2%), from Lusaka Urban District (78.5%) and <30 years at enrolment (46.5%) (Table 1). Most KPIF beneficiaries were reached after the start of our intervention, largely in 2022 (56.4%). Two‐thirds (67.0%) of beneficiaries were provided services at a community venue—significantly more than at static sites (*p* < 0.001)—in line with our intervention.

**Table 1:** Key Population Investment Fund programme beneficiary demographics, September 2019–March 2023 (*N* = 38,307)

Characteristic	FSW *n* = 19,989 *n* (%)	MSM *n* = 13,348 *n* (%)	PWID *n* = 3642 *n* (%)	TG *n* = 1328 *n* (%)	Total *N* = 38,307 *n* (%)	*p*‐value
**Intervention status**						
Pre‐intervention	2864 (14.3)	1303 (9.8)	482 (13.2)	93 (7.0)	4742 (12.4)	<0.001
Post‐intervention	17,125 (85.7)	12,045 (90.2)	3160 (86.8)	1235 (93.0)	33,565 (87.6)	
**Reported biological sex at birth**						
Male	0 (0)	13,348 (100)	3349 (92.0)	846 (63.7)	17,543 (45.8)	<0.001
Female	19,989 (100)	0 (0)	293 (8.0)	482 (36.3)	20,764 (54.2)	
**Age band (years)**						
15−19	779 (3.9)	419 (3.1)	95 (2.6)	49 (3.7)	1342 (3.5)	<0.001
20−24	3798 (19.0)	2599 (19.5)	670 (18.4)	281 (21.2)	7348 (19.2)	
25−29	4641 (23.2)	3323 (24.9)	827 (22.7)	340 (25.6)	9131 (23.8)	
30−34	4370 (21.9)	2989 (22.4)	801 (22.0)	348 (26.2)	8508 (22.2)	
35−39	3038 (15.2)	1753 (13.1)	539 (14.8)	167 (12.6)	5497 (14.3)	
40−44	1934 (9.7)	1169 (8.8)	349 (9.6)	89 (6.7)	3541 (9.2)	
45−50	976 (4.9)	674 (5.0)	220 (6.0)	37 (2.8)	1907 (5.0)	
50+	453 (2.3)	422 (3.2)	141 (3.9)	17 (1.3)	1033 (2.7)	
**Programme districts**						
Chilanga	1660 (8.3)	439 (3.3)	282 (7.7)	13 (1.0)	2394 (6.3)	<0.001
Chongwe	1685 (8.4)	851 (6.4)	405 (11.1)	38 (2.9)	2979 (7.8)	
Kafue	1485 (7.4)	1014 (7.6)	344 (9.4)	28 (2.1)	2871 (7.5)	
Lusaka Urban	15,159 (75.8)	11,044 (82.7)	2611 (71.7)	1249 (94.1)	30,063 (78.5)	
**Programme enrolment year**						
2019 (Q3−4)	562 (2.8)	321 (2.4)	11 (0.3)	74 (5.6)	968 (2.5)	<0.001
2020	1716 (8.6)	620 (4.6)	326 (9.0)	3 (0.2)	2665 (7.0)	
2021	474 (2.4)	262 (2.0)	101 (2.8)	11 (0.8)	848 (2.2)	
2022	10,610 (53.1)	8208 (61.5)	1807 (49.6)	993 (74.8)	21,618 (56.4)	
2023 (Q1−2)	6627 (33.2)	3937 (29.5)	1397 (38.4)	247 (18.6)	12,202 (31.9)	
**Service delivery setting**						
Static site	7175 (35.9)	3688 (27.6)	1515 (41.6)	271 (20.4)	12,649 (33.0)	<0.001
Venue	12,814 (64.1)	9660 (72.4)	2127 (58.4)	1057 (79.6)	25,658 (67.0)	

Abbreviations: FSW, female sex worker; MSM, men who have sex with men; PWID, person who injects drugs; Q, programme quarter; TG, transgender person.

**Figure 2:**
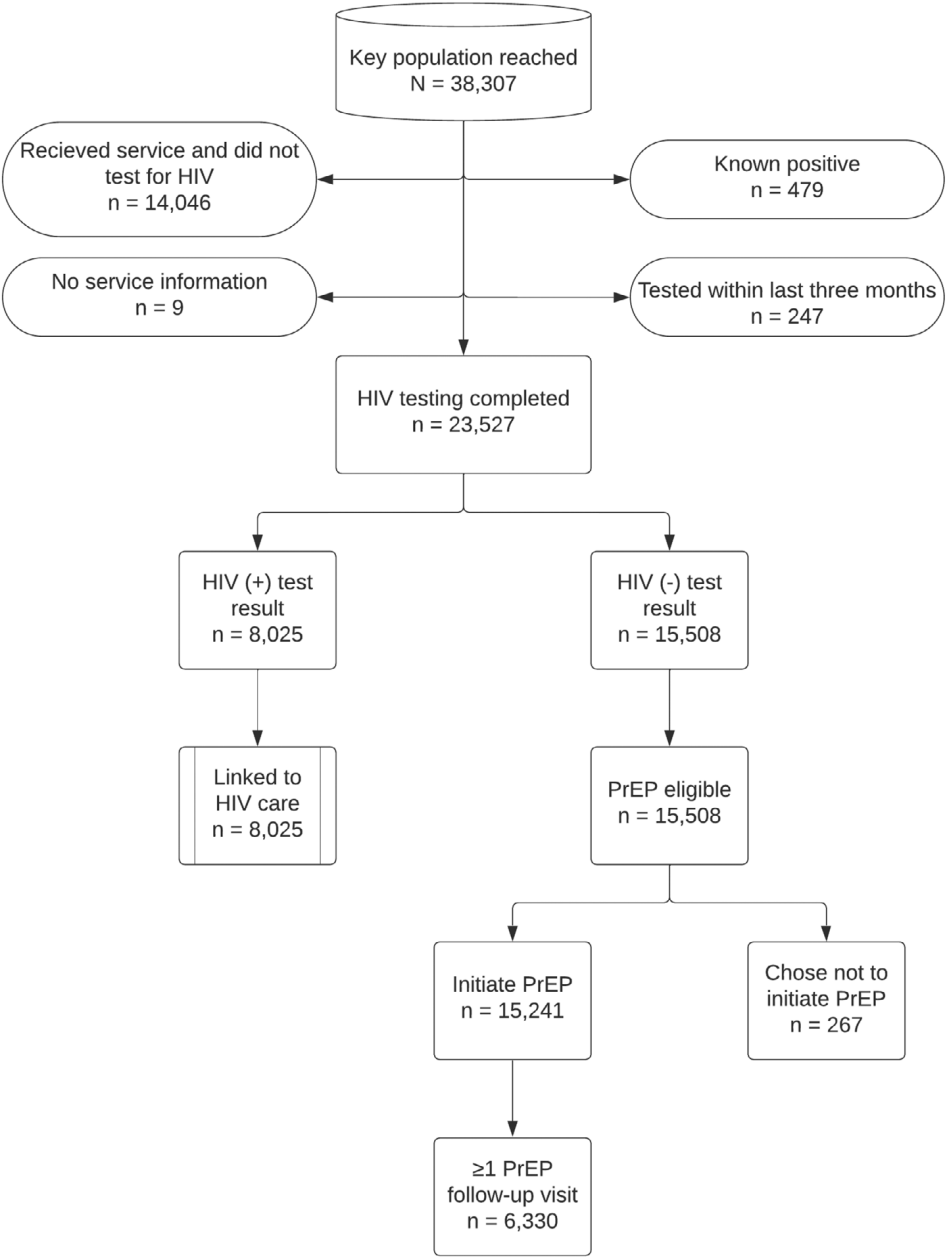
**Key Population Investment Fund programme beneficiary flow**. Abbreviations: KP, key population; PrEP, pre‐exposure prophylaxis.

Of the 38,307 KPIF beneficiaries, 23,527 (61.4%) received HIV testing services and 14,046 (36.7%) received ≥1 HIV prevention service and did not test for HIV. Of those tested, 15,508 (65.9%) tested negative. Of these, 100% were PrEP eligible per MOH guidelines and 15,241 (98.3%) initiated PrEP (Figure 2).

### 3.3 PrEP eligibility and uptake

**Figure 3:**
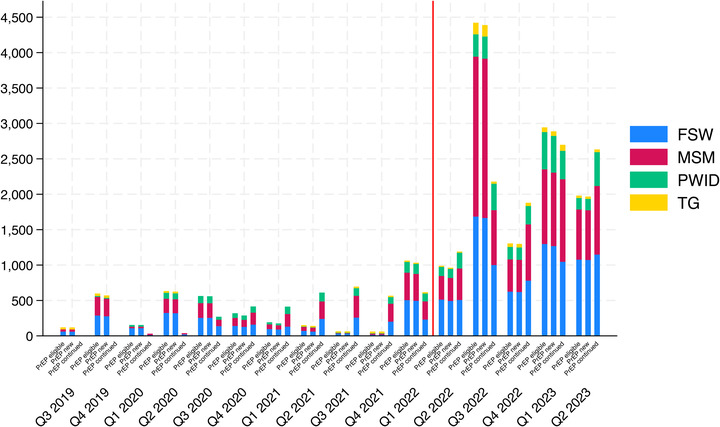
**PrEP uptake by key population group and year in the Key Population Investment Fund Programme**. Abbreviations: FSW, female sex worker; MSM, men who have sex with men; PrEP, pre‐exposure prophylaxis; PWID, people who inject drugs; Q, quarter; TG, transgender people.

The numbers of KP with a documented HIV‐negative test result and eligible for PrEP per national guidelines increased from 2019 through 2023, despite a notable decrease in 2021 because of the COVID‐19 delta wave that temporarily interrupted services. The absolute number of KP who initiated PrEP increased markedly following the start of the venue‐based service delivery (red line in Figure 3). Across all programme quarters and KP groups, PrEP uptake among those eligible was consistently >90%.

PrEP uptake increased significantly following the introduction of our venue‐based PrEP intervention in December (Q4) of 2021, with a higher proportion initiating PrEP after (i.e. Q1 2022 through Q2 2023) versus before (i.e. Q3 2019 through Q4 2021) the intervention (98.7% vs. 96.5%, *p* < 0.001). While the frequency of PrEP initiations was increasing before our intervention, the observed difference remained statistically significant after adjusting for time‐related trends in an interrupted time series sensitivity analysis (*p* = 0.014) (Figure S1).

### 3.4 PrEP use continuity

KPIF began tracking PrEP follow‐up and re‐initiation visits in 2020. We observed a quarterly increasing trend in the absolute number of PrEP continuation visits (i.e. either follow‐up or re‐initiation) for all KP groups (Figure 3). In most quarters, the number of PrEP continuation visits in the quarter matched or exceeded the number of new PrEP initiations in the preceding quarter.

**Figure 4:**
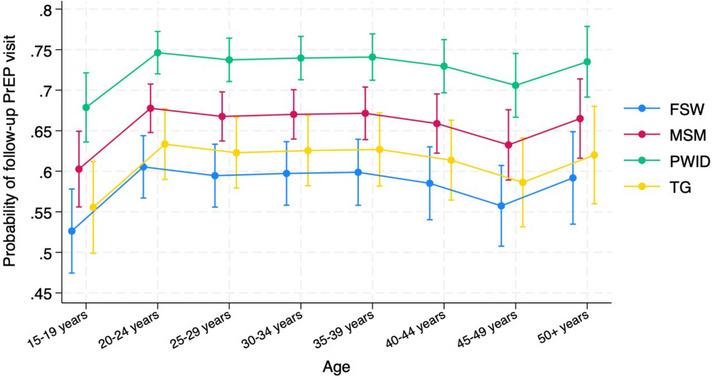
**Marginal probability and accompanying 95% confidence intervals of PrEP follow‐up within 6 months of initiation**. Abbreviations: FSW, female sex worker; MSM, men who have sex with men; PrEP, pre‐exposure prophylaxis; PWID, people who inject drugs; TG, transgender people.

The proportion of KP who had ≥1 continuation visit for a PrEP refill within 6 months of initiation remained stable pre‐intervention (47.2%, 95% confidence interval [CI]: 45.4%, 49.1%) compared to post‐intervention (46.7%, 95% CI: 45.7%, 47.6%). The probability of having a PrEP continuation visit within 6 months varied significantly by KP group and age band, with PWID having the highest estimated marginal probability (Figure 4). Young KP ages 15–19 years had a lower estimated probability of a PrEP follow‐up visit than KP ≥20 years old.

### 3.5 PrEP cascades

**Figure 5:**
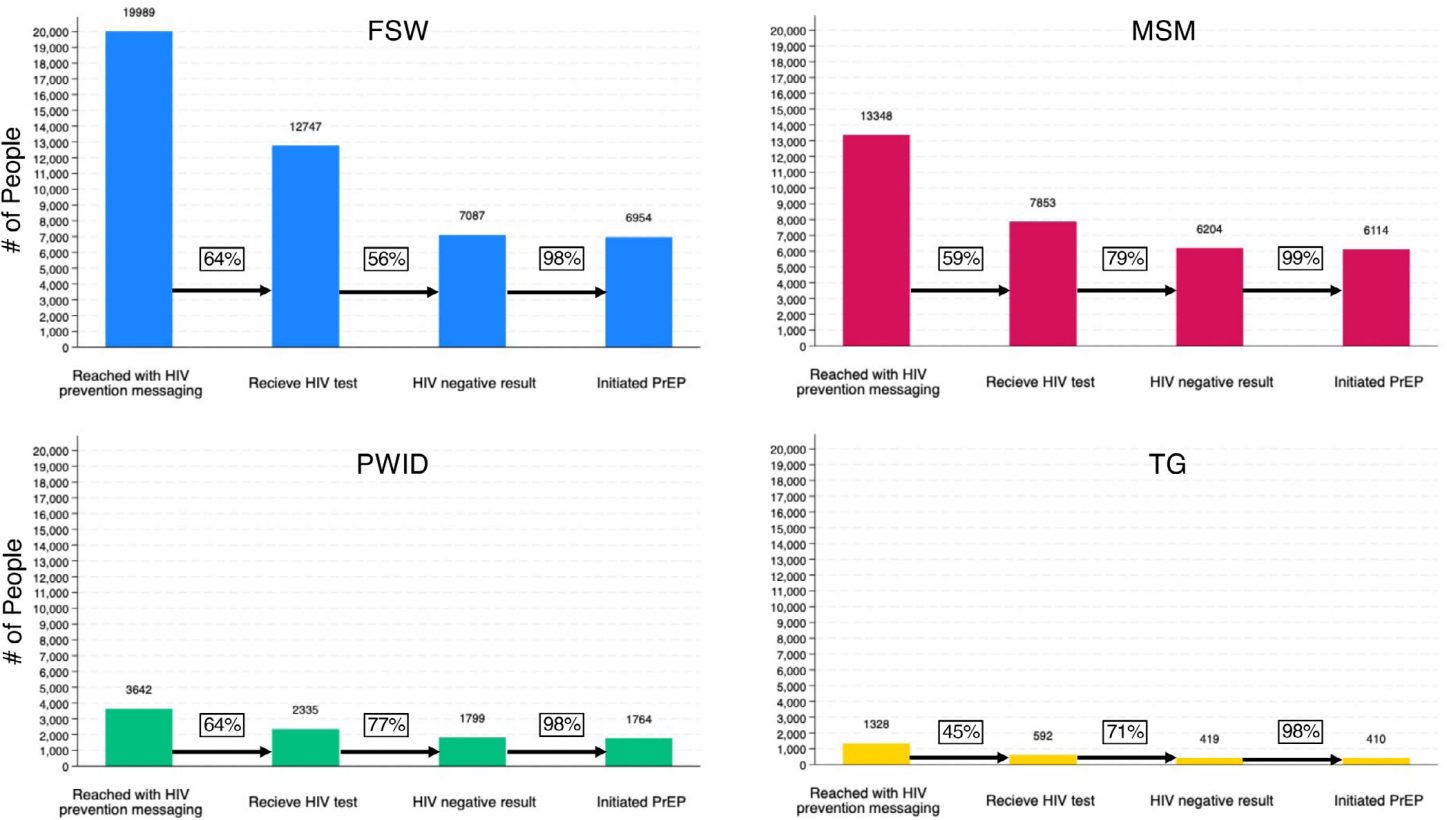
**PrEP prevention cascades for Key Population Investment Fund programme beneficiaries**. Abbreviations: FSW, female sex worker; MSM, men who have sex with men; PrEP, pre‐exposure prophylaxis; PWID, people who inject drugs; TG, transgender people.

Since KPIF inception, more FSWs have been screened, found PrEP eligible and initiated PrEP than any other KP group, followed closely by MSM (Figure 5). Among KP reached, the proportion testing for HIV ranged between 45% and 64%. In all KP groups, PrEP uptake among those found HIV negative and PrEP‐eligible was ≥98%.

### 3.6 PrEP equity—PrEP initiations among KP versus general population

At programme inception, most PrEP initiations occurred in the general population. The share of PrEP initiations among KP increased significantly after (60.0%; 95% CI: 59.3%, 60.6%) versus before (24.8%; 95% CI: 24.0%, 25.6%) introduction of venue‐based PrEP provision (*p* < 0.001), with KP accounting for most PrEP initiations by the end of the evaluation period (Figure S2).

### 3.7 Data use, knowledge translation and stakeholder engagement

At the site level, KPIF staff held daily meetings to discuss programme implementation and monthly meetings to review PrEP uptake, retention and seroconversions. These meetings were led by KP CSO leaders and included CIDRZ and MoH staff. At the district level, KPIF held regular quarterly data review meetings—with equal frequency before and after our intervention—resulting in over 60 such meetings between September 2019 and March 2023. Finally, at the programme level, KPIF held joint programme review meetings twice yearly to collectively identify strategies to improve and sustain programme performance. Through these meetings, granular quality improvement plans were made to sustain early successes and overcome identified challenges.

## 4 DISCUSSION

We illustrate how the practical application of a Programme Science cycle, informed by the effective programme coverage framework, can produce advancements in PrEP service delivery for populations that have been historically underserved in the HIV response. By leveraging an extensive programme platform, robust routine data sources and a novel programme intervention, we were able to address a programmatically meaningful research question related to PrEP uptake, continuity and equity for KP. We describe an increase in PrEP initiation and continuation visits for all KP groups following the introduction of PrEP in venue‐based settings and expansion to urban community‐based safe spaces. The increase in PrEP utilization we observed fits with other data from Zambia and the region showing greater PrEP availability with recent scale‐up [20, 21]. Our experience aligns with models incorporating community‐based approaches that increased PrEP uptake and continuity [22, 23, 24, 25]. The use of differentiated PrEP services co‐created with KP beneficiaries, particularly with PWID and TG who remain marginalized and hard‐to‐reach in our setting, remains critical if HIV acquisition risk is to be abated.

Programme Science offers a useful lens from which to make context‐specific insights into the nature of, and opportunities for, innovation in routine HIV prevention programming. Using elements of the effective programme coverage framework, we estimated the required coverage for the KPIF programme based on PEPFAR targets and assessed other dimensions of programme coverage from routinely collected data. We demonstrated that a community‐based PrEP intervention enabled increased PrEP utilization among diverse KP groups. That being the case, gaps in the PrEP cascade remain, with our data revealing a need for greater HIV testing uptake and support for PrEP continuation.

Our approach enabled the specification of programme components that likely contributed to the favourable programmatic effects observed. First, empowering KP CSOs to plan, mobilize and monitor programme implementation, with technical support for clinical service delivery, was associated with rapid PrEP scale‐up, which has been shown to be critical in other PrEP programmes [25, 26, 27]. Second, securing buy‐in from stakeholders beyond the beneficiary communities, such as venue owners and patrons [28], was necessary to ensure support for venue‐based PrEP delivery. This facilitated the beneficiary‐responsive decentralized approach used, resulting in successes despite implementation challenges like COVID‐19 [29]. Third, we developed standardized data collection tools for monitoring and evaluation through a broad consultative process with stakeholders, including KP CSOs [30].

Leveraging the robust KPIF programme platform and strategy, we followed a series of pragmatic steps, here presented as a Programme Science cycle. At the programme design phase, we utilized a community participatory approach with KP CSOs to identify the most pressing question for programme improvement and posed it *a priori* as an evaluation question tied to relevant outcomes. We focused our evaluation metrics on reach, adoption and equity—all established outcomes drawn from implementation science [31, 32, 33]. As findings for these outcomes emerged, they were fed back to KP CSOs for knowledge translation, iterative quality improvement and advocacy. At programme and district levels, data showing limited PrEP continuation, particularly among FSW and young KP, accelerated KP CSO community‐led monitoring and catalysed formation of PrEP support groups and a peer navigator‐PrEP beneficiary pairing programme to support early PrEP persistence and adherence. The relatively low proportion of PrEP clients observed continuing PrEP at 6 months fits with other reports showing low retention among KP in SSA [34], and in Thailand where a KP CSO programme achieved PrEP retention of 43.9% at 12 months for MSM and TG women [35]. Further work is needed to identify pragmatic strategies to address PrEP continuation barriers, and to align programmatic definitions of PrEP continuity with more empirical, risk‐aligned definitions [36].

Despite Programme Science's potential impact for advancing HIV prevention, several challenges limited its usefulness, rigour and relevance. In our experience, these included difficulties in obtaining district‐level population‐based size estimates to establish required coverage [8], and data missingness and misspecification in the routine record. For example, it was difficult to determine the precise duration of PrEP refills for some clients due to incomplete pharmacy data. To mitigate these challenges, regular data use, quality assurance and data science approaches are warranted.

Novel application of analytical methods embedded within the programme, like polling booth surveys [37] and sampling‐based approaches [38], borrowed from other disciples, can enhance Programme Science rigour. Finally, additional resources are required to build data use capacity among KP CSOs for community‐led monitoring.

Our study had additional methodological limitations. First, it is possible that there was a misclassification of KP type, for example, if KPIF counsellors misidentified a TG woman as MSM. Second, our data for general population members could have included KP who did not openly disclose being a KP member due to stigma or social desirability bias. Third, in keeping with a pragmatic programme science approach harnessing routine data, we used an observational pre‐post study design without a control group. This design may have inadequately adjusted for confounding. Third, and finally, the observed effects could be attributed, at least in part, to other explanations independent from our main programmatic innovation, such as KP CSO capacity building and empowerment.

These methodological limitations notwithstanding, Programme Science can help generate actionable insights for improving HIV prevention programming. In our example of venue‐based PrEP delivery, we observed a lower estimated probability of a follow‐up visit for FSW compared to other KP groups, which fits with data from the region highlighting the PrEP continuity challenges faced by this highly mobile population [34]. Venue‐based PrEP distribution allowed prevention services to be delivered in less stigmatizing settings [39], closer to where FSWs work, which may have enabled better‐than‐expected PrEP continuation compared to facility‐based settings. Further Programme Science inquiry is needed to identify ways to improve continued PrEP delivery for FSW, as well as for the most hidden KP, including TG and PWID.

## 5 CONCLUSIONS

We applied Programme Science principles and tenants of the effective programme coverage framework to demonstrate how decentralizing HIV prevention services to KP venues enabled successful public‐sector, community‐based PrEP delivery beyond the reach of traditional facility‐based approaches. The CIDRZ KPIF approach is being taken to scale in Zambia with support from PEPFAR, MOH and KP CSOs who must be adequately resourced to continue acting on lessons from Programme Science to sustain recent population‐level gains in HIV prevention.

## COMPETING INTERESTS

None of the authors have any competing interests.

## AUTHORS’ CONTRIBUTIONS

MM and CM led KPIF programme management. All authors contributed to PrEP service delivery as part of the implementing team and/or technical assistance. IS and MEH conceptualized the manuscript. IS led the writing and coordination with support from MEH and MM. KC was responsible for data collection and data cleaning. JMP led the data analysis with support from KC. All authors reviewed and contributed to the writing.

## FUNDING

The study was supported by the U.S. Centre for Disease Control and Prevention, Cooperative Agreement NU2GGH001920.

## Supporting information


**Figure S1**: Interrupted time series plot with level change regression model

**Figure S2**: Proportion of all PrEP initiations who are key population members for all Key Population Investment Fund‐supported catchment areas

## Data Availability

The data that support the findings of this study are available on request from the corresponding author. The data are not publicly available due to privacy or ethical restrictions.
