# Supplementary material for: Programme science in action: lessons from an observational study of HIV prevention programming for key populations in Lusaka, Zambia
Source: J Int AIDS Soc. 2024 Jul 10;27(Suppl 2):e26237. doi: 10.1002/jia2.26237 (PMC11233926; doi:10.1002/jia2.26237)
Supplement: Supplementary file 1 — Figure S1: Interrupted time series plot with level change regression model [file JIA2-27-e26237-s001.pdf]

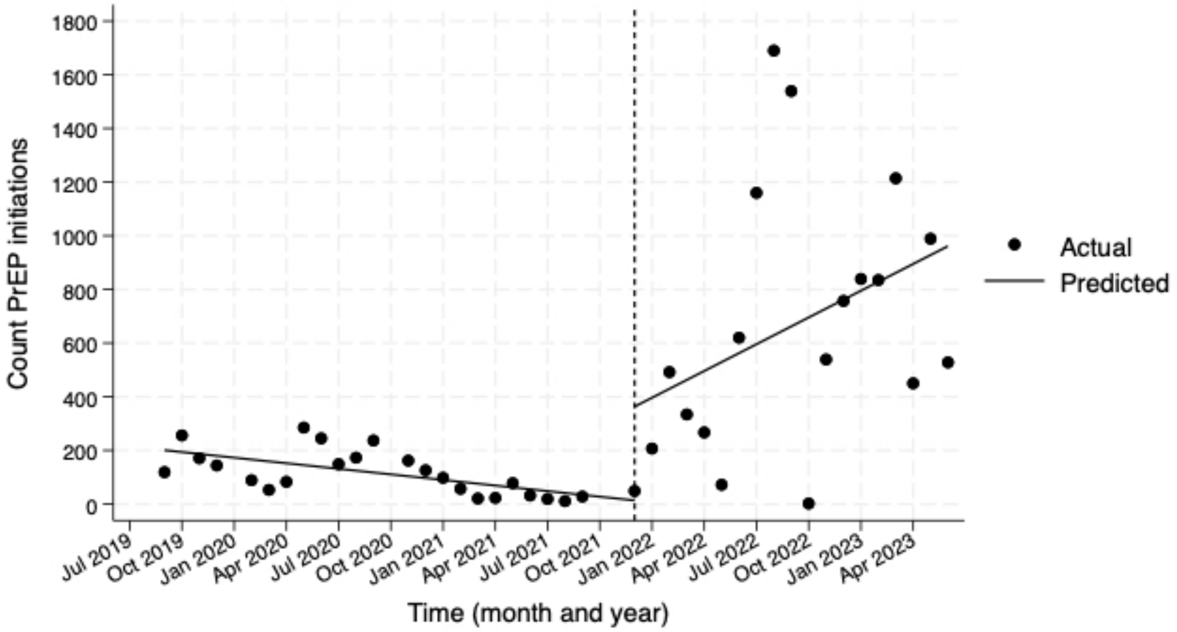

Figure S1. Interrupted time series with level change regression model. Solid lines reflects the predicted trend based on the model. The dashed vertical line reflects the time period of intervention introduction on 1 December, 2021. PrEP – pre-exposure prophylaxis.
