# Supplementary material for: Programme science in action: lessons from an observational study of HIV prevention programming for key populations in Lusaka, Zambia
Source: J Int AIDS Soc. 2024 Jul 10;27(Suppl 2):e26237. doi: 10.1002/jia2.26237 (PMC11233926; doi:10.1002/jia2.26237)
Supplement: Supplementary file 2 — Figure S2: Proportion of all PrEP initiations who are key population members for all Key Population Investment Fund‐supported catchment areas [file JIA2-27-e26237-s002.pdf]

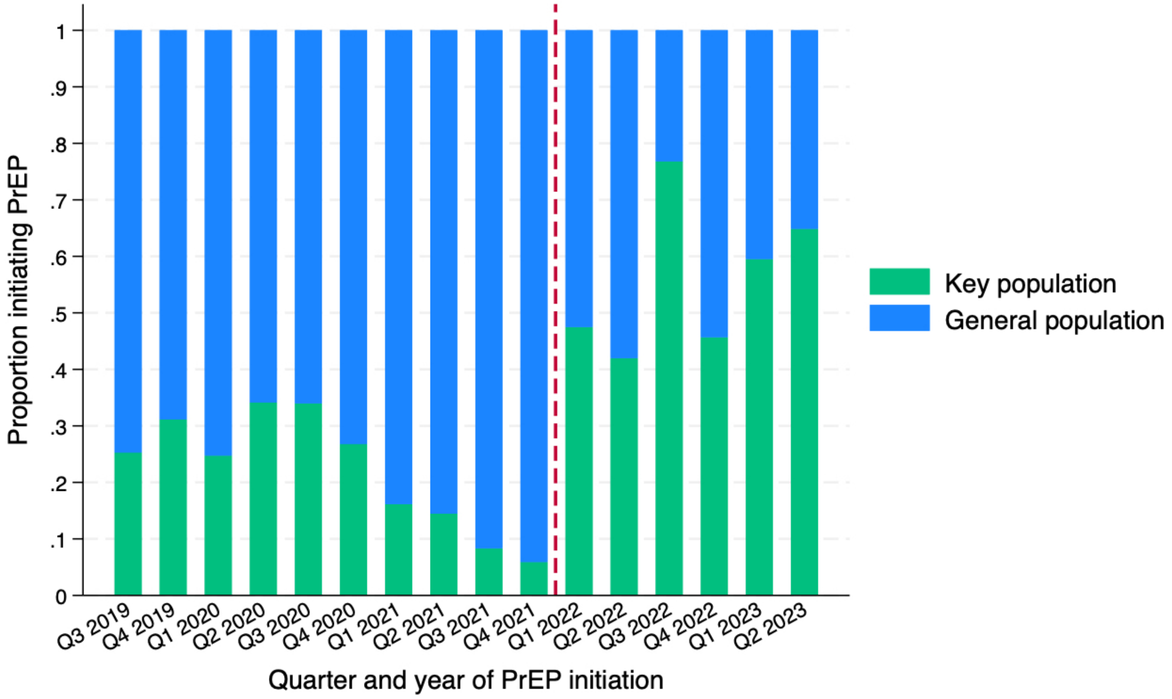

Figure S2. Proportion of all PrEP initiations who are key population members for all Key Population Investment Fund-supported catchment areas (dashed red line indicates before versus after intervention introduction). PrEP – pre-exposure prophylaxis.
